# Impact of pericardial fluid glucose level and computed tomography attenuation values on diagnosis of malignancy-related pericardial effusion

**DOI:** 10.1186/s12872-021-02091-6

**Published:** 2021-06-03

**Authors:** Takashi Nakamura, Mana Okune, Masakazu Yasuda, Heitaro Watanabe, Masafumi Ueno, Kenji Yamaji, Kazuki Mizutani, Takashi Kurita, Gaku Nakazawa

**Affiliations:** Division of Cardiology, Department of Internal Medicine, Kindai University Faculty of Medicine, 377-2 Ohno-Higashi, Osakasayama, 589-8511 Japan

**Keywords:** Pericardial effusion, Pericardial fluid glucose level, Malignancy, CT attenuation values

## Abstract

**Background:**

We evaluated malignancy according to the characteristics of pericardial fluid in symptomatic Japanese patients undergoing pericardiocentesis and computed tomography (CT).

**Methods:**

This was a retrospective, single-center, observational study of 125 symptomatic patients undergoing pericardiocentesis. The patients were classified into two groups: a malignancy group and a non-malignancy group, according to the primary disease and cytology of the pericardial effusion (PE). We compared the pericardial fluid sample and CT measurements between both groups.

**Results:**

All patients were diagnosed as having exudative PE by Light’s criteria. PE with malignant cells was demonstrated in 76.8% of the malignancy group patients. Pericardial to serum lactate dehydrogenase (LDH) ratio > 0.6, as one of Light’s criteria, was associated with malignancy (*p* = 0.017). Lower serum brain natriuretic peptide (BNP) concentration was also associated with malignancy (BNP: 126.9 ± 89.8 pg/ml vs 409.2 ± 97.7 pg/ml, malignancy vs non-malignancy groups, respectively; *p* = 0.037). A significant difference was observed in pericardial fluid glucose level between the malignancy and non-malignancy groups (pericardial fluid glucose: 78.24 ± 48.29 mg/dl vs 98.41 ± 44.85, respectively; *p* = 0.048). Moreover, CT attenuation values (Hounsfield units (HU)) tended to be higher in the malignancy group vs the non-malignancy group (22.7 [interquartile range (IQR), 17.4–26.0] vs 17.4 [IQR, 13.7–26.4], respectively; *p* = 0.08). The sensitivity and specificity of pericardial fluid glucose level ≤ 70 mg/dl and CT attenuation values > 20 HU were 40.9% and 89.6%, respectively, in the malignancy group. The positive- and negative predictive values of pericardial fluid glucose level ≤ 70 mg/dl and CT attenuation values > 20 HU were 85.7% and 50.0%, respectively, in the malignancy group. Pericardial fluid glucose level ≤ 70 mg/dl and CT attenuation values > 20 HU were cutoff values associated with malignancy (*p* = 0.012).

**Conclusions:**

Lower pericardial fluid glucose level with higher CT attenuation values may suggest malignancy-related PE.

**Supplementary Information:**

The online version contains supplementary material available at 10.1186/s12872-021-02091-6.

## Background

The normal pericardial sac contains up to 50 ml of pericardial fluid as a plasma ultrafiltrate. Pericardial effusion (PE), which is classified according to composition, distribution, and volume, results from many diseases [1]. In patients with PE, the prevalence of malignancy or infection ranges from 15 to 50% [2, 3], and malignant PE can be diagnosed by cytology. In addition to imaging and pericardial fluid analysis, the clinical course of the underlying disease can contribute to the diagnosis of malignancy-related PE. In particular, large PE is a common manifestation in malignant disease, and patients with malignant PE have a poor prognosis because of advanced-disease stage [4, 5]. In cases without pre-existing cancer, malignancy must be excluded because PE may be the first manifestation of malignancy. Some studies reported PE attributed to unknown causes at a rate of 10%–20% [5, 6]. It may be difficult to diagnose malignant PE even if cytology or epicardial and pericardial biopsy are performed [7]. A previous study of pericardial fluid analysis showed that diseases with exudative PE had lower pericardial fluid glucose levels compared with diseases with transudative PE [8]. However, the relationship between malignancy-related PE and non-malignant PE evaluated using computed tomography (CT) to analyze the pericardial fluid is unknown. Therefore, we examined malignancy according to the characteristics of the pericardial fluid and CT measurements in patients with symptoms associated with PE undergoing pericardiocentesis.

## Methods

### Patients

We enrolled 125 consecutive symptomatic patients undergoing pericardiocentesis between April 2010 and April 2020 as a retrospective observational study in a single institution.

Twenty-eight patients without pericardial fluid sampling and CT data were excluded because of out-of-hospital cardiopulmonary arrest, Stanford type A aortic dissection, and blow-out rupture in acute myocardial infarction as an intra-procedural complication. All patients who did not undergo thoracic CT scans because of cardiac shock were diagnosed on echocardiography in the emergency room. Therefore, 97 patients were finally enrolled in this study. The patients were classified into two groups: a malignancy group and a non-malignancy group. The malignancy group constituted patients with known existing malignancy or documented malignant cells in drained PE.

### Blood samples and pericardial fluid samples

According to the patients’ medical records, blood samples were collected for routine care approximately 24 h after the first drainage. The following were evaluated in the drained pericardial fluid: pH, total protein, lactate dehydrogenase (LDH), albumin, glucose, adenosine deaminase (ADA), hyaluronic acid, hemoglobin (Hb), and hematocrit. Collected pericardial fluid was cytologically diagnosed with malignant cells or non-malignant cells by two pathologists. Pericardial fluid testing was performed in 88 patients, and cytological analysis was performed in 94 patients. Patients were diagnosed with exudative PE according to Light’s criteria, which constitute a pericardial fluid-to-serum total protein ratio > 0.5 and a pericardial fluid-to-serum LDH ratio > 0.6 or two-thirds of the upper limit of the normal (UNL) serum LDH value [9].

### CT protocol and measurement

**Fig. 1:**
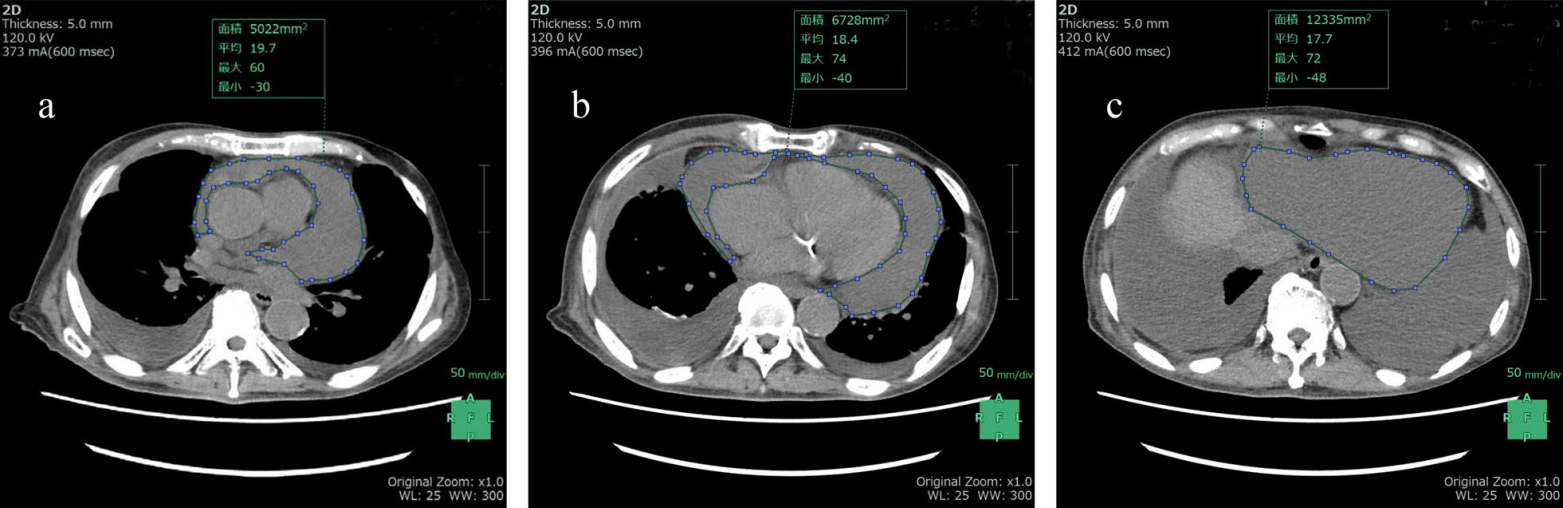
Measuring CT attenuation values in pericardial effusion. Two-dimensional images in three axial views: **a** upper slice: bifurcation of the pulmonary trunk, **b** middle slice: four-chamber heart view, **c** lower slice: upper edge of the liver. Both computed tomography (CT) attenuation values (in Hounsfield units (HU)) and pericardial effusion area were measured by plotting around the pericardial effusion in the three axial slices. The respective results were **a** 5022 mm^2^, 19.7 HU; **b** 6728 mm^2^, 18.4 HU; and **c** 12,335 mm^2^, 17.7 HU

Non-enhanced CT scans before drainage were performed within 24 h of admission using a 64-multidetctor CT scanner (Discovery HD 750; GE Healthcare, Milwaukee, WI, USA). Eighty-one patients were evaluated, with 5-mm slice thickness, gantry rotation speed of 350 ms, 64 × 0.625-mm collimation, tube voltage of 120 kV, and effective tube current of 325–750 mA. Axial images were transferred to an available workstation and analyzed using SYNAPSE VINCENT software (Fujifilm™ Co., Tokyo, Japan). The CT attenuation values in the PE were calculated and analyzed by SYNAPSE VINCENT [10]. In addition to a previous method that was determined by the region of interest (ROI), CT attenuation values and pericardial area were measured by plotting around the PE using a two-dimensional (2D) image analysis system in three axial view slices as follows: (1) upper slice: bifurcation of the pulmonary trunk, (2) middle slice: four-chamber heart view, and (3) lower slice: upper edge of the liver (Fig. 1) [11]. The average CT attenuation value from the three slices was calculated as Hounsfield units (HU).

### Statistical analysis

Categorical variables were compared using the χ^2^ test for proportions and the unpaired t-test or analysis of variance for continuous variables, as appropriate. The linearity of the relationship between two variables was assessed by linear regression analysis. Correlation analysis was performed with Pearson’s correlation coefficient. Parameters with non-normal distributions were analyzed using the Mann–Whitney U test. *p* < 0.05 was considered significant, and results are expressed as the mean ± standard deviation (SD). CT attenuation values were measured by two cardiologists, and interobserver agreement was evaluated. The cardiologists independently analyzed the images and discussed the CT measurements. Interobserver reliability was calculated as intraclass correlation coefficient, and interobserver agreement was calculated as the k coefficient. In the case of interobserver disagreement, we selected CT attenuation values that were close to the CT attenuation values measured by a third cardiologist. All analyses were performed using JMP version 11.0.

## Results

### Baseline clinical characteristics

The baseline characteristics of the 97 subjects are shown in Table 1; the majority were male and > 65 years of age. Approximately 10% of the patients had previously taken antiplatelet agents, but few patients had received prior anticoagulation therapy. The most frequent etiology in patients undergoing pericardiocentesis was malignant disease (58.8%), and among these patients, 59.6% had lung cancer (Table 2). Pericardial fluid with malignant cells: positive-cytology was demonstrated in 76.8% of the patients in the malignancy group.

**Table 1:** The patients’ baseline characteristics

Characteristic	n = 97
Age (years)	66.1 ± 14.2
Men (%)	58 (59.8)
Medications (%)	
Prior antiplatelet	10 (10.3)
Prior novel oral anticoagulants	6 (6.2)
Prior warfarin	5 (5.2)
Symptom (%)	
Dyspnea	84 (86.6)
Cough	15 (15.5)
Anorexia	10 (10.3)
Chest pain	9 (9.3)
Syncope	5 (5.2)
Primary drained pericardial effusion (ml)	550.87 ± 262.31
Etiology (%)	
Malignancy	57 (58.8)
Collagen vascular diseases	8 (8.2)
Idiopathic	7 (7.2)
Infection	5 (5.1)
Post-myocardial infarction	4 (4.1)
Uremia	4 (4.1)
Iatrogenic	3 (3.1)
Post-surgery	3 (3.1)
Chronic graft-versus-host disease	3 (3.1)
Radiation	1 (1.0)
Dissection	1 (1.0)
Hypothyroidism	1 (1.0)

The baseline clinical characteristics of all subjects are shown

**Table 2:** Etiologies in the malignancy group. Among patients with malignant disease, patients with lung cancer constituted the highest percentage

Malignancy	n = 57
Lung cancer	34 (59.6)
Gastrointestinal carcinoma	7 (12.3)
Malignant lymphoma	3 (5.3)
Renal carcinoma	3 (5.3)
Breast carcinoma	2 (3.5)
Gynecological carcinoma	2 (3.5)
Pleural mesothelioma	1 (1.8)
Hemangiosarcoma	1 (1.8)
Primary cancer of unknown type	4 (7.0)

### Blood samples (Table 3)

**Table 3:** Comparison between the malignancy and non-malignancy groups

Serum component	Malignancy group	Non-malignancy group	*p*
CRP (mg/dl)	5.33 ± 4.37	4.54 ± 6.70	0.488
eGFR (ml/min/1.73 m^2^)	60.56 ± 31.42	45.97 ± 29.08	0.024
Total protein (g/dl)	6.08 ± 0.60	6.06 ± 1.27	0.889
LDH (U/L)	543.35 ± 767.60	357.98 ± 186.54	0.138
Albumin (g/dl)	3.01 ± 0.46	3.11 ± 0.75	0.399
Glucose (mg/dl)	142.20 ± 61.03	134.10 ± 53.05	0.518
Hemoglobin (g/dl)	11.01 ± 1.77	11.11 ± 1.60	0.766
Hematocrit (%)	33.29 ± 5.61	34.24 ± 4.84	0.391
BNP (pg/ml)	126.89 ± 183.35	409.18 ± 868.67	0.037
Pericardial fluid component			
Ph	7.39 ± 0.20	7.48 ± 0.12	0.069
Total protein (g/dl)	5.02 ± 0.80	4.72 ± 1.19	0.166
LDH (U/L)	2032.10 ± 3522.33	1029.58 ± 1561.52	0.106
Albumin (g/dl)	2.66 ± 0.53	2.61 ± 0.70	0.661
Glucose (mg/dl)	78.24 ± 48.29	98.41 ± 44.85	0.048
ADA (U/L)	37.59 ± 47.26	29.34 ± 37.66	0.389
Hyaluronic acid (ng/ml)	31,975.9 ± 27,282.7	27,194.6 ± 28,745.9	0.438
Hemoglobin (g/dl)	3.83 ± 2.22	5.15 ± 5.19	0.422
Hematocrit (%)	14.79 ± 6.73	16.74 ± 21.36	0.788
Variable			
Pericardial fluid/serum ratio			
Total protein ratio	0.83 ± 0.14	0.78 ± 0.15	0.127
LDH ratio	5.23 ± 7.69	2.59 ± 2.71	0.047
Glucose ratio	0.57 ± 0.34	0.83 ± 0.45	0.005
Pericardial fluid/Serum total protein ratio > 0.5	49 (100%)	38 (97.4%)	0.260
Pericardial fluid/Serum LDH ratio > 0.6	47 (94.0%)	29 (76.3%)	0.017
Pericardial fluid LDH > 2/3 UNL	49 (98.0%)	32 (86.5%)	0.036
Cytology, positive	43 (76.8%)	−	−

Lower serum BNP and lower pericardial fluid glucose level were associated with malignancy

Values are expressed as mean ± standard deviation

*CRP* C-reactive protein, *eGFR* estimated glomerular filtration rate, *LDH* lactate dehydrogenase, *BNP* brain natriuretic protein, *ADA* adenosine deaminase, *UNL* upper limit of normal

No significant differences in serum C-reactive protein (CRP), total protein, LDH, albumin, glucose, and Hb were observed between the malignancy and non-malignancy groups. In contrast, estimated glomerular filtration rate (eGFR) was significantly higher in patients with vs without malignancy (60.56 ± 31.42 ml/min/1.73 m^2^ vs 45.97 ± 29.08 ml/min/1.73 m^2^, respectively; *p* = 0.024). Serum brain natriuretic peptide (BNP) levels were significantly lower in patients with vs without malignancy (126.89 ± 183.35 pg/ml vs 409.18 ± 868.67 pg/ml, respectively; *p* = 0.037).

Pericardial fluid samples, pericardial/serum ratio, Light’s criteria, and cytology (Table 3).

**Fig. 2:**
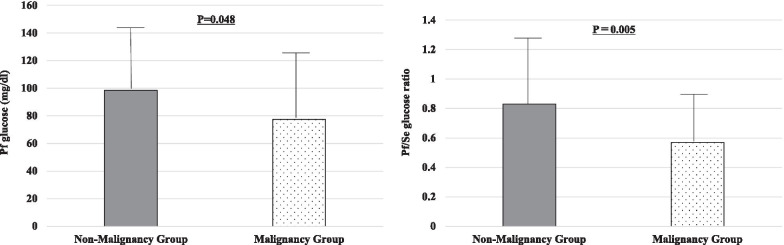
Comparison of the Pf glucose level and Pf/Se glucose ratio between the groups. Pericardial fluid glucose levels and pericardial fluid/serum glucose ratio were significantly lower in the malignancy group than in the non-malignancy group. *Pf* pericardial fluid, *Se* serum

No significant differences in pericardial fluid pH levels, total protein, LDH, albumin, ADA, and hyaluronic acid were observed between the two groups. Pericardial fluid glucose levels were significantly lower in patients with vs without malignancy (78.24 ± 48.29 vs 98.41 ± 44.85, respectively; *p* = 0.048) (Fig. 2).

Malignant cells in the pericardial fluid were demonstrated in 76.8% of the patients in the malignancy group. All patients were diagnosed as having exudative PE by Light’s criteria. Of the three items in Light’s criteria, LDH ratio > 0.6 and pericardial LDH > 2/3 of the UNL were associated with malignancy (*p* = 0.017 and *p* = 0.036, respectively). The pericardial fluid-to-serum LDH ratios were significantly higher in patients with malignancy than in those without malignancy (5.23 ± 7.69 vs 2.59 ± 2.71, respectively; *p* = 0.047). The pericardial fluid-to-serum glucose ratios were significantly lower in patients with vs without malignancy (0.57 ± 0.34 vs 0.83 ± 0.45, respectively; *p* = 0.005) (Fig. 2).

### CT measurements

The interobserver correlation for determining CT attenuation values indicated good agreement [interclass correlation coefficient, 0.965 (95% confidence interval (CI), 0.946–0.977)]. The interobserver agreement for determining CT attenuation values also indicated good agreement [κ coefficient, 0.82].

**Fig. 3:**
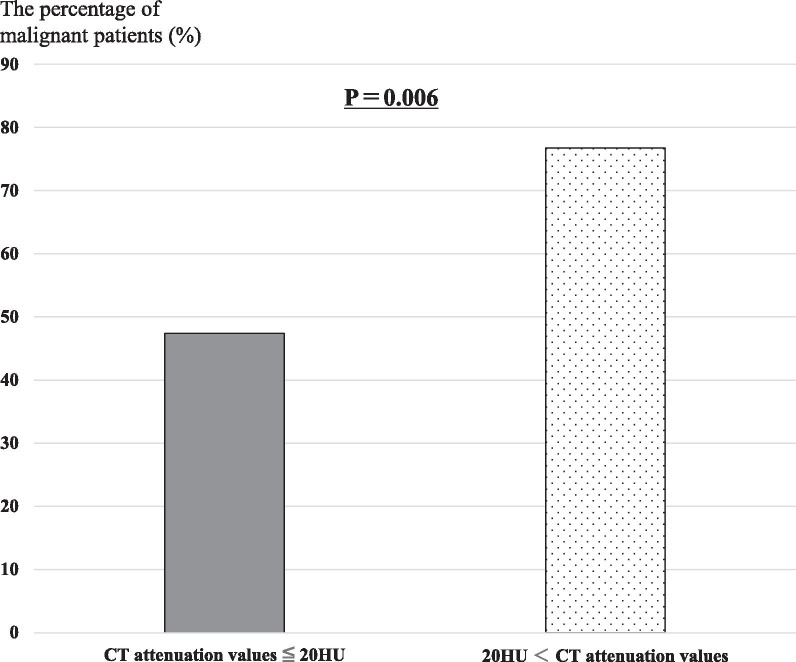
Correlation with CT attenuation values and the percentage of malignant patients. Comparison between the percentage of malignant patients with computed tomography attenuation values ≤ 20 Hounsfield units (HU) and the percentage of malignant patients with computed tomography (CT) attenuation values > 20 HU

CT attenuation values (HU) tended to be higher in the malignancy group compared with the non-malignancy group (22.7 [interquartile range (IQR), 17.4–26.0] vs 17.4 [IQR, 13.7–26.4], respectively). However, the p-value was 0.08; therefore, no significant difference in CT attenuation values was observed between the malignancy and non-malignancy groups. In contrast, CT attenuation values > 20 HU were associated with malignancy (*p* = 0.006) (Fig. 3).

The relationship between pericardial fluid samples and CT measurements.

**Fig. 4:**
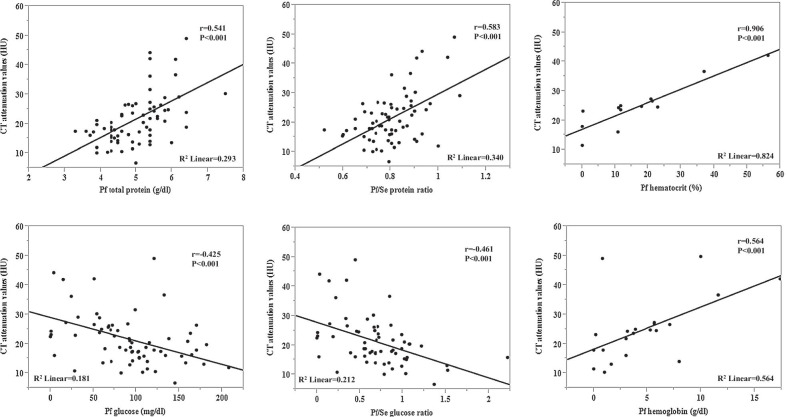
Correlation between CT attenuation values and pericardial fluid parameters. The pericardial fluid parameters were: total protein level, protein ratio, glucose level, glucose ratio, hematocrit, and hemoglobin. Each dot represents one patient; the straight line represents the best fit line obtained by linear regression analysis. *Pf* pericardial fluid, *Se* serum, *HU* Hounsfield units, *CT* computed tomography

**Fig. 5:**
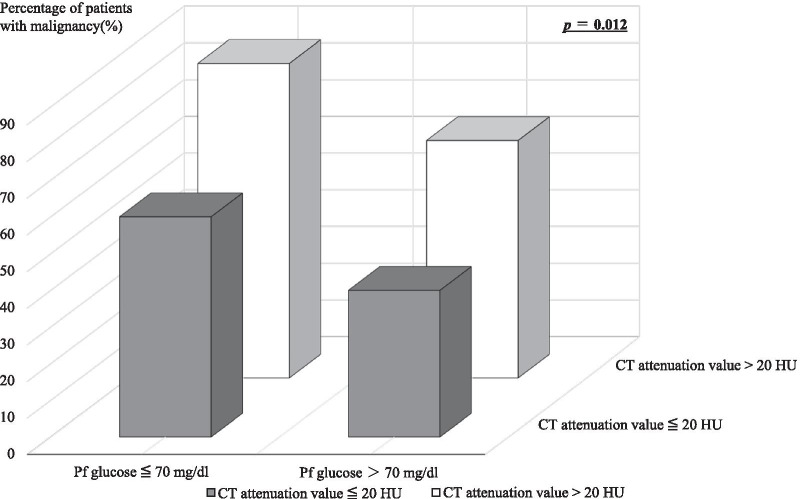
Relationship between pericardial fluid glucose or CT attenuation values and malignancy vs non-malignancy. Pericardial fluid glucose ≤ 70 mg/dl and CT attenuation values > 20 HU were cutoff values associated with malignancy. *Pf* pericardial fluid, *CT* computed tomography

The relationship between pericardial fluid measurements and CT attenuation values is shown in Fig. 4. CT attenuation values were strongly correlated with pericardial fluid hematocrit (*r* = 0.908). CT attenuation values were also correlated with pericardial fluid glucose level (*r* =  − 0.425), protein level (*r* = 0.541), glucose ratio (*r* =  − 0.461), and protein ratio (*r* = 0.583). The sensitivity and specificity of pericardial fluid glucose level ≤ 70 mg/dl and CT attenuation values > 20 HU were 40.9% and 89.6%, respectively, in the malignancy group. The positive- and negative predictive values of pericardial fluid glucose level ≤ 70 mg/dl and CT attenuation values > 20 HU were 85.7% and 50.0%, respectively, in the malignancy group. Pericardial fluid glucose ≤ 70 mg/dl and CT attenuation values > 20 HU were cutoff values associated with malignancy (Fig. 5). Additionally, pericardial fluid glucose ≤ 70 mg/dl and CT attenuation values > 20 HU were also cutoff values associated with positive-cytology patients (see Additional file 1: Fig. S1).

## Discussion

To the best of our knowledge, the current study is the first to investigate malignancy-related PE by adding the characteristics of CT images to the pericardial fluid examination. We found that lower pericardial fluid glucose level and higher CT attenuation values were related to PE owing to malignancy. In particular, pericardial fluid analysis in patients undergoing pericardiocentesis is performed routinely, but the results of this analysis are not effectively used in clinical practice.

Malignant PE is a serious manifestation associated with a poor prognosis [4, 5]. However, PE may be the first manifestation of malignancy in some cases without pre-existing cancer; therefore, malignant PE must be considered, even if the cytology is negative. In the current study, the combination of lower pericardial fluid glucose level and higher CT attenuation values was a useful tool for differentiating malignancy-related PE from non- malignancy-related PE.

Strobbe et al. evaluated the etiologies of PE and distinguished radiation therapy for malignancy as a cause of PE [12]. Moreover, the authors reported that bone marrow transplantation was an etiology in the idiopathic group [12]. Additionally, Sullivan et al. reported that chronic graft-versus-host disease (GVHD) presented as an immune reaction after bone marrow transplantation, similar to the mechanism in autoimmune diseases [13]. Therefore, non-recurrent cancer patients with prior radiation therapy or chronic GVHD were included in our non-malignancy group.

In the current study, the number of patients in the malignancy group was higher than the number in the non-malignancy group, and approximately 60% of the patients in the malignancy group had lung cancer. While the percentage of patients with an idiopathic etiology in our study was 7.2%, one study in Western Asia and another in Africa reported PE attributed to unknown causes at rates of approximately 10%–15% [6, 14]. In contrast, a study in Western Europe and another in North America reported that approximately 80%–90% of patients with PE were classified as having an idiopathic etiology [2, 15]. Distinguishing malignant PE from non-malignant PE has important implications regarding prognosis. However, two previous studies reported that 44.9%–92% of patients with malignancy often demonstrated malignant cells in PE [8, 16]. As a result of cytology by repeat drainage, the current study showed that 76.8% of patients in the malignancy group demonstrated malignant cells in the pericardial fluid. Standard pericardial cytology does not necessarily exclude malignant PE; therefore, it is necessary to detect neoplasia using blood samples, pericardial fluid analysis, and imaging modalities, such as positron-emission tomography-CT (PET-CT). Cytology-negative patients in the malignancy group in this study were defined as patients who had untreatable recurrent or metastatic carcinoma.

Although pericardial fluid analysis is performed routinely, the results of this analysis are not effectively used owing to a lack of specificity. Meyers et al. reported that pericardial fluid glucose level was lower in patients with exudates than in those with transudates, and there was a significant overlap in the etiology of PE between patients with transudates vs exudates [8]. All cases in our study, in both the malignancy and non-malignancy groups, were diagnosed as having exudative PE by Light’s criteria. No significant differences in pericardial fluid LDH levels were observed between the groups, while LDH ratio > 0.6 and pericardial LDH > 2/3 of the UNL were associated with malignancy. Ben-Horin et al. demonstrated pericardial fluid LDH level > 2.4 times the serum level and mean pericardial fluid protein level 0.6 times the serum level in patients undergoing elective open surgery without a history of pericardial diseases [17]. Despite these findings, a recent study showed that patients with malignancy had lower PE LDH levels than patients without malignancy, and another study showed that malignant patients had lower PE glucose levels compared with patients with heart failure-related effusion [16, 18]. Furthermore, Ben-Horin et al. demonstrated a mean pericardial fluid glucose level of 133 mg/dl and mean pericardial fluid-to-serum glucose ratio of 1.0 in patients without a history of pericardial diseases [17]. In the current study, pericardial fluid glucose levels and pericardial fluid-to-serum glucose ratio were significantly lower in the malignancy group than in the non-malignancy group. In an additional analysis, pericardial fluid glucose levels were significantly lower in positive-cytology patients than in negative-cytology patients. However, neither pericardial fluid glucose levels nor the pericardial fluid-to-serum glucose ratio in our study was helpful for differentiating the malignancy group from the non-malignancy group because the areas under the receiver operating characteristic (ROC) curve were < 0.7. Based on these results, the cutoff value for pericardial fluid glucose level under the ROC curve for differentiating the malignancy group from the non-malignancy group was 70 mg/dl. Pericardial fluid glucose level may decrease in diseases such as tuberculosis, rheumatism, and inflammatory diseases in non-malignancy patients, and serum glucose level can change secondary to various factors. The results of Gram’s stain and acid-fast bacteria stain of pericardial fluid were negative in this study. Additionally, no significant differences in the rates of diabetes mellitus and hypoglycemic agents were observed between the groups regarding pericardial fluid glucose levels. In contrast, CT attenuation values may be useful in determining the etiology of PE. We measured CT attenuation values in PE areas lager than the area in the ROI and used the former as the CT attenuation value. Previous studies reported that CT attenuation values were positively correlated with pleural and pericardial total protein levels and the pleural and pericardial/serum protein ratio [11, 19]. Moreover, Rifkin et al. demonstrated that CT attenuation values were strongly correlated with pericardial fluid hematocrit [20]. Our study showed that CT attenuation values were strongly correlated with pericardial fluid hematocrit, and that these values also had a good correlation with pericardial fluid glucose and protein levels, and glucose ratio and protein ratio. CT attenuation values from 20 to 60 HU indicated exudate in hypothyroidism, purulent pericarditis, or malignancy [21]. The median CT attenuation values in both groups was > 20 HU for exudates, while the cutoff value was determined to be 20 HU according to a previous report indicating that patients with CT attenuation values > 20 HU had proteinaceous or hemorrhagic PE [22]. In the current study, the cutoff value for CT attenuation under the ROC curve for differentiating the malignancy group from the non-malignancy group was 19.7 HU; approximately 20 HU. As a result, CT attenuation values > 20 HU were associated with malignancy.

In our study, PE for differentiating the malignancy group from the non-malignancy group was evaluated by the combination of pericardial fluid glucose level and CT attenuation values. Pericardial fluid glucose level ≤ 70 mg/dl and CT attenuation values > 20 HU had 40.9% sensitivity and 89.6% specificity for discriminating the malignancy group from the non-malignancy group. Additionally, our study demonstrated that CT attenuation values had a good correlation with pericardial fluid glucose level. According to our results, the combination of pericardial fluid glucose ≤ 70 mg/dl and CT attenuation values > 20 HU might be associated with malignancy in PE. In addition, this study showed that this combination was associated with positive-cytology patients.

Recent studies showed that pericarditis related to malignancy, except for pericardial infiltration of malignant cells and radiation therapy, was associated with antineoplastic agents, such as tyrosine kinase inhibitors and immune checkpoint inhibitors [23, 24]. Although we attempted to exclude PE related to antineoplastic agents, it was difficult to demonstrate the effect of these therapies in negative-cytology patients in the malignancy group because this was a diagnosis of exclusion. In future, we must pay attention to antineoplastic agent use in patients with malignancy and PE.

The strength of this study is that we evaluated diagnostic procedures that are readily available in clinical practice. Pericardial fluid analysis and CT imaging are simple and routine tools to diagnose malignancy-related PE. Pericardial fluid glucose level and CT attenuation values can be obtained quickly; therefore, this effective method should be used in clinical practice.

### Study limitations

There are several limitations to our study. First, the size of the non-malignancy group was small because this was a single-institution study. Second, the malignancy group had higher proportions of patients with lung cancer. The retrospective nature of this study is another limitation.

## Conclusions

Lower pericardial fluid glucose level in addition to higher CT attenuation values may suggest malignancy-related PE. In patients with pericardial fluid glucose ≤ 70 mg/dl and CT attenuation values > 20 HU, malignant disease as the cause of PE must be excluded by other examinations and imaging modalities, even if there are no malignant cells in pericardial fluid cytology.

## Supplementary Information

**Additional file 1: Fig. S1**. Relationship between pericardial fluid glucose or CT attenuation values and positive cytologyvs negative cytology. Pericardial fluid glucose ≤ 70 mg/dl and CT attenuation values > 20 HU were cutoffvalues associated with positive cytology. *Pf*pericardial fluid, *CT* computed tomography.

## Data Availability

The datasets used and/or analyzed during the current study are available from the corresponding author on reasonable request.

## Abbreviations

CT: Computed tomography
PE: Pericardial effusion
LDH: Lactate dehydrogenase
BNP: Brain natriuretic peptide
HU: Hounsfield units
IQR: Interquartile range
ADA: Adenosine deaminase
UNL: Upper limit of normal
ROI: Region of interest
2D: Two-dimensional
CRP: C-reaction protein
Hb: Hemoglobin
eGFR: Estimated glomerular filtration rate
PET-CT: Positron-emission tomography-CT
ROC: Receiver operating characteristic
