# Supplementary material for: Impact of pericardial fluid glucose level and computed tomography attenuation values on diagnosis of malignancy-related pericardial effusion
Source: BMC Cardiovasc Disord. 2021 Jun 3;21:272. doi: 10.1186/s12872-021-02091-6 (PMC8176742; doi:10.1186/s12872-021-02091-6)
Supplement: Supplementary file 1 — Additional file 1: Fig. S1. Relationship between pericardial fluid glucose or CT attenuation values and positive cytologyvs negative cytology. Pericardial fluid glucose ≤ 70 mg/dl and CT attenuation values > 20 HU were cutoffvalues associated with positive cytology. Pfpericardial fluid, CT computed tomography. [file 12872_2021_2091_MOESM1_ESM.pptx]

## Slide 1
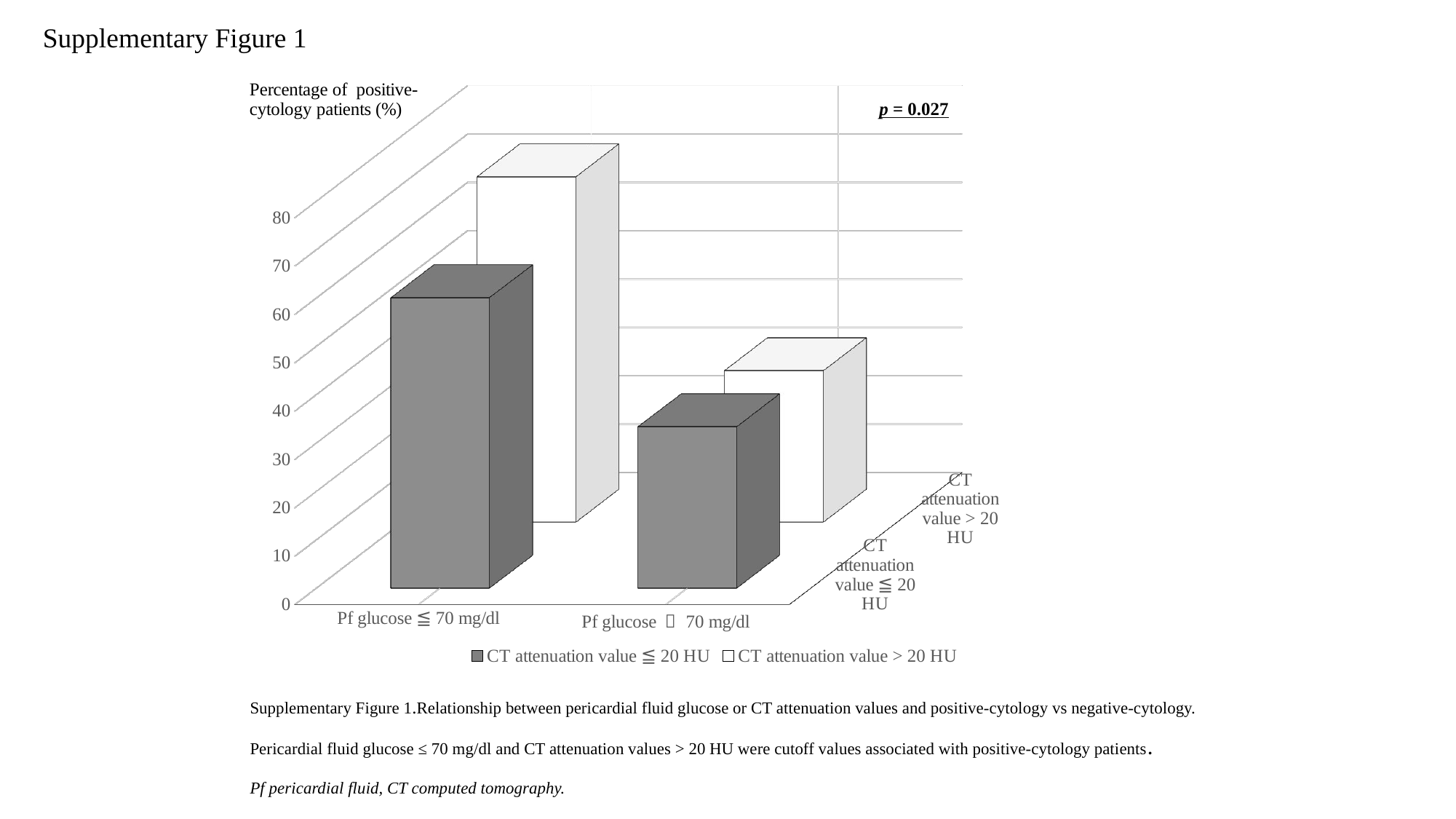

Supplementary Figure 1
[unsupported chart]
Supplementary Figure 1.Relationship between pericardial fluid glucose or CT attenuation values and positive-cytology vs negative-cytology.
Pericardial fluid glucose ≤ 70 mg/dl and CT attenuation values > 20 HU were cutoff values associated with positive-cytology patients.
Pf pericardial fluid, CT computed tomography.
